# Supplementary material for: Mechanisms governing the pioneering and redistribution capabilities of the non-classical pioneer PU.1
Source: Nat Commun. 2020 Jan 21;11:402. doi: 10.1038/s41467-019-13960-2 (PMC6972792; doi:10.1038/s41467-019-13960-2)
Supplement: Supplementary file 7 — Source data [file 41467_2019_13960_MOESM7_ESM.zip › Source_Data/FigureS2/FigureS2B_MotifScanOutput/KmeansCluster9/homerResults/motif13.similar.html]

motif13

## Information for motif13

A
T
G
C
C
T
G
A
T
A
G
C
G
C
A
T
A
C
G
T
A
T
G
C
C
G
A
T
A
T
C
G
G
A
T
C
G
C
A
T
A
G
C
T
G
A
C
T
  
Reverse Opposite:  

C
T
G
A
T
C
G
A
C
G
T
A
C
T
A
G
T
A
G
C
C
G
T
A
A
T
C
G
C
G
T
A
C
G
T
A
A
T
C
G
G
A
C
T
A
T
C
G
  

|  |  |
| --- | --- |
| p-value: | 1e-33 |
| log p-value: | -7.697e+01 |
| Information Content per bp: | 1.747 |
| Number of Target Sequences with motif | 95.0 |
| Percentage of Target Sequences with motif | 4.17% |
| Number of Background Sequences with motif | 370.5 |
| Percentage of Background Sequences with motif | 0.80% |
| Average Position of motif in Targets | 101.8 +/- 43.0bp |
| Average Position of motif in Background | 102.2 +/- 69.6bp |
| Strand Bias (log2 ratio + to - strand density) | 0.4 |
| Multiplicity (# of sites on avg that occur together) | 1.00 |
| Motif File: | file (matrix) reverse opposite |

### Similar de novo motifs found

|  |  |  |  |  |  |  |  |
| --- | --- | --- | --- | --- | --- | --- | --- |
| Rank | Match Score | Redundant Motif | P-value | log P-value | % of Targets | % of Background | Motif file |
| 1 | 0.921 | T A G C A T G C G C T A T A G C A G C T A G C T A T G C G C A T A T G C A G C T G A C T G C A T G A C T G A C T | 1e-29 | -67.765137 | 3.25% | 0.54% | motif file (matrix) |
| 2 | 0.976 | C G T A T C G A C G T A T C A G T A G C C G T A C T A G T G C A C T G A T A C G C G A T A T C G G A C T | 1e-26 | -61.455622 | 5.10% | 1.47% | motif file (matrix) |
| 3 | 0.939 | G T C A T G A C C G A T C A G T T A G C G C A T A T C G A T G C G C A T A G C T C A G T | 1e-19 | -43.774689 | 5.62% | 2.19% | motif file (matrix) |
